# Reciprocal positive effects on parasitemia between coinfecting haemosporidian parasites in house sparrows

**DOI:** 10.1186/s12862-022-02026-5

**Published:** 2022-06-02

**Authors:** Luz Garcia-Longoria, Sergio Magallanes, Xi Huang, Anna Drews, Lars Råberg, Alfonso Marzal, Staffan Bensch, Helena Westerdahl

**Affiliations:** 1 Department of Anatomy, Cellular Biology and Zoology, University of Extremadura, E-06071 Badajoz, Spain; 2 Molecular Ecology and Evolution Lab, Department of Biology, Lund University, Sölvegatan 37, SE-22362 Lund, Sweden; 3 MOE Key Laboratory for Biodiversity Science and Ecological Engineering, College of Life Science, Beijing Normal University, 100875 Beijing, China

**Keywords:** Co-infection, House sparrow, Plasmodium, Haemoproteus

## Abstract

**Background:**

Hosts are often simultaneously infected with several parasite species. These co-infections can lead to within-host interactions of parasites, including mutualism and competition, which may affect both virulence and transmission. Birds are frequently co-infected with different haemosporidian parasites, but very little is known about if and how these parasites interact in natural host populations and what consequences there are for the infected hosts. We therefore set out to study *Plasmodium* and *Haemoproteus* parasites in house sparrows *Passer domesticus* with naturally acquired infections using a protocol where the parasitemia (infection intensity) is quantified by qPCR separately for the two parasites. We analysed infection status (presence/absence of the parasite) and parasitemia of parasites in the blood of both adult and juvenile house sparrows repeatedly over the season.

**Results:**

*Haemoproteus passeris* and *Plasmodium relictum* were the two dominating parasite species, found in 99% of the analyzed Sanger sequences. All birds were infected with both *Plasmodium* and *Haemoproteus* parasites during the study period. Seasonality explained infection status for both parasites in the adults: *H. passeris* was completely absent in the winter while *P. relictum* was present all year round. Among adults infected with *H. passeris* there was a positive effect of *P. relictum* parasitemia on *H. passeris* parasitemia and likewise among adults infected with *P. relictum* there was a positive effect of *H. passeris* parasitemia on *P. relictum* parasitemia. No such associations on parasitemia were seen in juvenile house sparrows.

**Conclusions:**

The reciprocal positive relationships in parasitemia between *P. relictum* and *H. passeris* in adult house sparrows suggests either mutualistic interactions between these frequently occurring parasites or that there is variation in immune responses among house sparrow individuals, hence some individuals suppress the parasitemia of both parasites whereas other individuals suppress neither. Our detailed screening of haemosporidian parasites over the season shows that co-infections are very frequent in both juvenile and adult house sparrows, and since co-infections often have stronger negative effects on host fitness than the single infection, it is imperative to use screening systems with the ability to detect multiple parasites in ecological studies of host-parasite interactions.

**Supplementary information:**

The online version contains supplementary material available at 10.1186/s12862-022-02026-5.

## Background

Hosts are frequently infected with more than one parasite species [1, 2]. As a consequence the co-infecting parasites share the host environment and in many cases also the host resources. It is noteworthy that co-infections often have stronger negative effects on host survival and fitness than single infections of each parasite species [3–6]. Birds are for example infected with a wide range of different avian haemosporidian parasites, i.e. malaria parasites (genus *Plasmodium*) and related parasites of the genus *Haemoproteus*. A single bird species can often be the host of ten different avian haemosporidian species [7, 8] and avian malaria can have severe fitness consequences for infected individuals [9].

During co-infections, the parasite species may interact in the host and these interactions can result in different consequences not only for the parasites but also for the host, i.e. varying severity of disease. There may for example be competition between the parasite species, where both parasite species suffer negative consequences in response to the presence of the other parasite species [10–12]. Facilitation can also occur, in which one or both species benefits from the interaction. In the commensal form of facilitation, one parasite benefits while the other is unaffected, and in mutualism, both parasites benefit from one another’s presence [13, 14].

In the case of malaria parasites, within-host interactions have gained considerable interest, both when studying natural occurrences of malaria infections in humans [15, 16] and also when using mice as a model species in the laboratory [17]. The human malaria parasites *Plasmodium falciparum* and *Plasmodium vivax* are often found in co-infections [18]. Studies of these two human malaria parasites have provided good examples of facilitation, where the presence of one parasite species favours the presence of the other species [13]. Facilitation occurs because *P. falciparum* is able to infect red blood cells (RBC) of all ages (mature and immature) leading to high levels of anaemia which force the host to generate new RBCs [19]. These new immature RBCs are the preferred resource of *P. vivax* and therefore *P. vivax* gains resources (immature RBCs) from the acute anaemia caused by the infection of *P. falciparum* [20]. However, other studies of malaria in humans have found different patterns of interactions between *P. falciparum* and *P. vivax*, suggesting that the within-host interactions are complex and context dependent [21–23]. It is challenging to study within-host interactions of haemosporidian parasites during co-infections in natural populations of wild animals, not only due to the need to recapture individuals but mainly because the occurrence of co-infections varies with many other temporal patterns over the season, such as vector abundance and infection status (presence/absence of the parasite in the blood) (reviewed in [24]).

Thus far co-infections of avian malaria have been studied experimentally in the laboratory by inoculation using different species of *Plasmodium* parasites. Palinauskas et al. (2011) [25] studied co-infections of the two avian malaria parasites *Plasmodium ashfordi* and *Plasmodium relictum* (specifically the lineage SGS1), and found that co-infections tended to have higher peak parasitemia than each of the two single infections, suggesting facilitation. In another experimental study Palinauskas et al. (2018) investigated co-infections of the avian malaria parasites *Plasmodium elongatum* and *P. relictum* (specifically the lineage SGS1) and again reported evidence of facilitation, specifically commensalism, with *P. elongatum* reaching higher *P. elongatum* parasitemia in the presence of *P. relictum* [26] but not vise versa.

Experimental set-ups and/or model host species have advantages in the study of co-infections but also limitations considering for example effects of natural diversity and artificial infections. We therefore set out to study co-infections of naturally occurring haemosporidian parasites which have had the opportunity to co-evolve in their natural host. House sparrows *Passer domesticus* in Spain are frequently infected with haemosporidian parasites [27–29], and provide an excellent study system for investigating co-infections of naturally occurring parasites in wild birds. The dominating haemosporidian species in house sparrows in southern Spain are *P. relictum* (lineages SGS1 and GRW11) and *Haemoproteus passeris* (specifically lineage PADOM05) [30]. *Plasmodium relictum* is an extreme host-generalist infecting more than 120 different bird species worldwide, whereas *H. passeris* is a host-specialist restricted to the genus *Passer* [31]. *Haemoproteus passeris* withdraw from the blood during the autumn and winter when it remains in internal organs, and thus cannot be detected by regular screening of blood samples and is considered a chronic and life-long infection. The prevalence of *P. relictum* in Spain is known to be highest in late spring and early autumn [7], and subsequently the two parasites *H. passeris* and *P. relictum* mainly have opportunity to interact in the bird blood during the summer months.

The parasitemia of haemosporidian parasites in bird blood is known to reach high levels during the acute phase of infection. The acute phase is followed by the chronic phase with low parasitemia, a level that in some cases can be below the detection limit. Moreover, in seasonal environments, some species of haemosporidian parasites completely withdraw from the blood, as mentioned above, and only remain in internal organs until conditions for transmission are favourable [32]. Accordingly, yearly relapses of infection can be measured in bird blood, though, relapses cannot be distinguished from secondary infections unless birds are kept under vector-controlled regimes. In our house sparrow study system relapses with comparatively low parasitemia are expected to be frequent in adult birds while novel infections with comparatively high infection intensities are expected to be frequent in juveniles.

We investigated the occurrence of haemosporidian parasites in the birds’ blood using repeated measures within individuals to monitor the infection status and parasitemia over the season in both adult and juvenile house sparrows. The birds were kept in outdoor aviaries, where they could be naturally infected with haemosporidian parasites by locally occurring vectors. Our main aim is to analyse and evaluate the consequences of co-infections, both considering infection status and parasitemia, with the distantly related haemosporidian parasites *P. relictum* and *H. passeris*. We expect the infection status of the parasites to vary over the season and we hypothesize that the parasitemia per parasite species will increase in co-infections.

## Results

### Infection status of *Plasmodium* and *Haemoproteus* in adults and juveniles

**Fig. 1:**
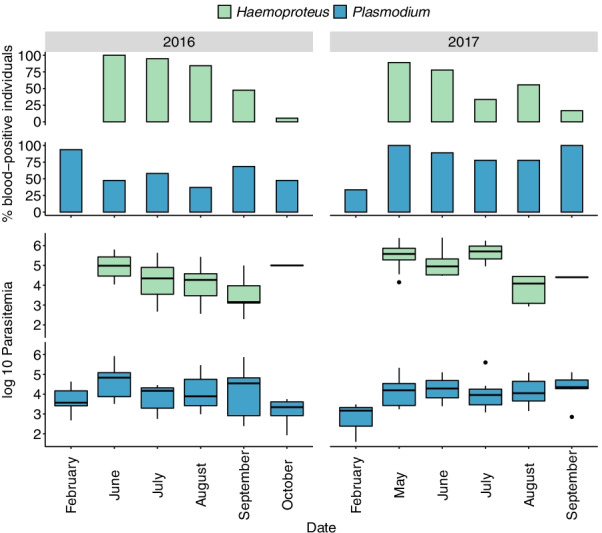
Seasonal changes in infection status and parasitemia in adult house sparrows. Changes in infection status (% blood-positive individuals) and infection intensity (log10 parasitemia, box plot where the bottom and top of the box indicates the first and third quartiles, the central line corresponds to the median, the whiskers represent the highest and lowest value within 1.5 * the interquartile range respectively) for *H. passeris* (green bars) and *P. relictum* (blue bars) in adult house sparrows, from February 2016 until September 2017 (the number of studied individuals varies slightly over time (N = 16 in February 2016, N = 19 in June 2016, N = 9 in February 2017 and N = 6 in September 2017), but the overall patterns are similar when only nine individuals are studied over the 18-month period (Additional file 3)

**Fig. 2:**
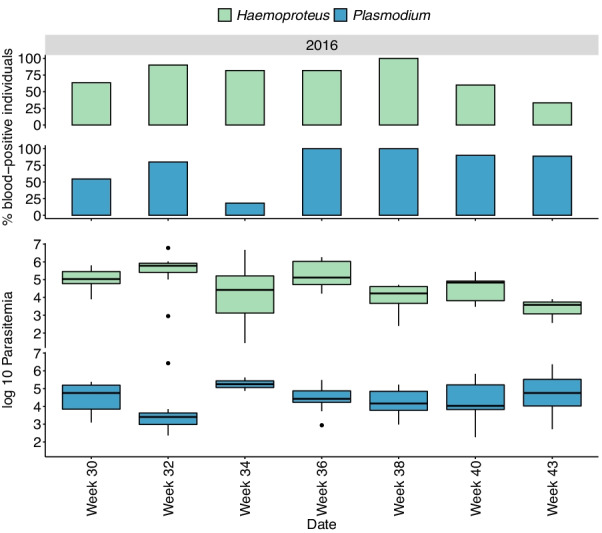
Seasonal changes in infection status and parasitemia in juvenile house sparrows. Changes in infection status (% blood-positive individuals) and infection intensity (log10 parasitemia, box plot where the bottom and top of the box indicates the first and third quartiles, the central line corresponds to the median, the whiskers represent the highest and lowest value within 1.5 * the interquartile range respectively) for *H. passeris* (green bars) and *P. relictum* (blue bars) in juvenile house sparrows, from July 2016 until October 2016 (week 30–43). The number of studied individuals is rather constant over time (N = 11 in week 30 and N = 9 in week 43)

Analyses by Sanger sequencing showed that 19 out of the 21 adult house sparrows were infected by both the haemosporidian parasites *P. relictum* (lineages SGS1 (N = 50) and GRW11 (N = 1)) and *H. passeris* (lineage PADOM05, N = 82, Additional file 1). The downstream analyses will be based on these 19 adults, which were infected with both parasites (Table 1, Additional file 2). *Plasmodium relictum* and *H. passeris* were detected in the birds’ blood at multiple sampling occasions (and frequently together) throughout the study period between February 2016 and September 2017 (Fig. 1). Likewise, analyses by Sanger sequencing combined with qPCR showed that 12 out of the 15 juveniles were infected by both the haemosporidian parasites *P. relictum* (lineage SGS1 (N = 12, Sanger sequences retrieved from seven different individuals) and *H. passeris* (lineage PADOM05, N = 50, Sanger sequences retrieved from 12 individuals, Additional file 1). Note however that another *Plasmodium* parasite, PAGRI02, was detected at two occasions in two juveniles in late autumn (Additional file 1). The downstream analyses will be based on 11 juveniles, which were infected with both *H. passeris* and *Plasmodium*, either SGS1 and/or PAGRI02, and sampled at least three times (Table 1, Additional file 2). *Plasmodium* and *H. passeris* were detected in the birds’ blood of juveniles at multiple time points (and frequently together) throughout the study period between July and October 2016 (Fig. 2).

**Table 1:** *Plasmodium* and *Haemoproteus* recorded as single and co-infections at 162 sampling occasions in adults and at 71 sampling occasions in juveniles (for further details see Additional file 2)

	Adults	Juveniles
*Haemoproteus*	32	12
*Plasmodium*	50	13
Both parasites	55	40
No parasites	25	6
Sum sampling occasions	162	71

### Infection status of both *H. passeris* and *P. relictum* vary over the season in adult birds

The infection status (presence/absence of the parasite in the blood) of *H. passeris* in adult sparrows differed between years (F = 11.38, df = 1, 136, P = 0.001) and months (F = 3.29, df = 6, 136, P = 0.0047, Fig. 1). The seasonal pattern was that *H. passeris* was absent in the winter, frequent in the spring and early summer, and then rare in late summer and autumn. The infection status of *H. passeris* was not affected by the *P. relictum* parasitemia (P = 0.53). Individual identity was included as a random factor in the model and had a significant effect on the infection status of *H. passeris* (Δ2LL = 19.2, P < 0.001). The infection status of *P. relictum* in adults was dependent on month (F = 456.04, df = 6, 131, P < 0.001, Fig. 1), but not on year (F = 0, df = 1, 131, P = 0.98), although there was an interaction between year and month (F = 3.51, df = 4, 131, P < 0.0093). *Haemoproteus passeris* parasitemia (P = 0.16) and individual identity (Δ2LL = 0) had no effect on the infection status of *P. relictum* in adult house sparrows. Co-infections were not more frequent than expected by chance (Fisher’s exact test, separate analyses for July, August, and September 2016 (N = 19 for each month): P = 0.141−0.579).

### Infection status of *H. passeris* and *Plasmodium* do not vary over the season in juveniles

The infection status of *H. passeris* in juveniles did not differ between weeks (F = 1.34, df = 6, 53, P = 0.25, Fig. 2), there was no effects of *Plasmodium* parasitemia on infection status of *H. passeris* (F = 2.50, df = 1, 59, P = 0.12) and no effect of the random factor individual identity (individual: Δ2LL = 2.96, P = 0.08). The infection status of *Plasmodium* in juveniles did not differ between weeks (F = 4.75, df = 6, 54, P = 0.062, Fig. 2), and there were no effects of *H. passeris* parasitemia (P = 0.82) or individual identity (individual: Δ2LL = 0) on infection status of *Plasmodium*.

### Reciprocal positive relationships between *P. relictum* and *H. passeris* parasitemia in adult house sparrows

**Fig. 3:**
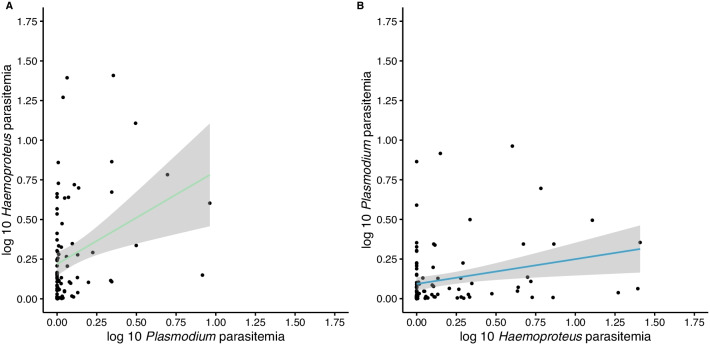
Reciprocal positive effects on parasitemia between *H. passeris* and *P. relictum* in adult house sparrows. The infection intensity (log 10 parasitemia) in adult house sparrows (**A**) blood positive with *H. passeris* (green) in relation with the infection intensity of *P. relictum* (log 10 parasitemia and also including non-infected individuals in this parameter, i.e. both blood positive and blood negative) and (**B**) blood positive with *P. relictum* (blue) in relation with the infection intensity of *H. passeris* (log 10 parasitemia and also including non-infected individuals in this parameter, i.e. both blood positive and blood negative). Both the positive correlations are significant and the grey area indicates the 95% confidence intervals (N = 19 adult house sparrows)

Our dataset did not allow us to compare the parasitemia in single and co-infection since most individuals carried both parasites. We therefore set out to analyse the consequences of co-infections by measuring the parasitemia per parasite in infected individuals. First, we investigated the consequences of the *P. relictum* parasitemia in individuals that had *H. passeris* in the blood. The parasitemia of *H. passeris* in adults (including only data points when an individual was blood-positive for *H. passeris* in the analysis) differed between years (F = 6.66, df = 1, 61, P = 0.012) and months (F = 4.49, df = 5, 61, P = 0.0015, Fig. 1). The parasitemia of *H. passeris* was positively correlated with the parasitemia of *P. relictum* (F = 4.71, df = 1, 61, P = 0.034, Fig. 3A). Thus, individuals that were infected with *H. passeris* had higher infection intensities when the *P. relictum* parasitemia was higher. The random factor individual had a significant effect on the *H. passeris* parasitemia (Δ2LL = 9.62, P = 0.002).

Second, we investigated the consequences of the *H. passeris* parasitemia in individuals that had *P. relictum* in the blood. The parasitemia of *P. relictum* in adults (including only data points when an individual was blood-positive for *P. relictum* in the analysis) differed between months (F = 2.44, df = 6, 78, P = 0.033, Fig. 1), but there was no effect of year (P > 0.13), and no interaction between year and month (P > 0.39). The parasitemia of *P. relictum* was positively correlated with the parasitemia of *H. passeris* (F = 4.96, df = 1, 78, P = 0.029, Fig. 3B), i.e., individuals that were infected with *P. relictum* had higher infection intensities when the *H. passeris* parasitemia was higher. No effect of individual was observed (individual: Δ2LL = 0).

### No relationships between *Plasmodium* and *H. passeris* parasitemia in juveniles

**Fig. 4:**
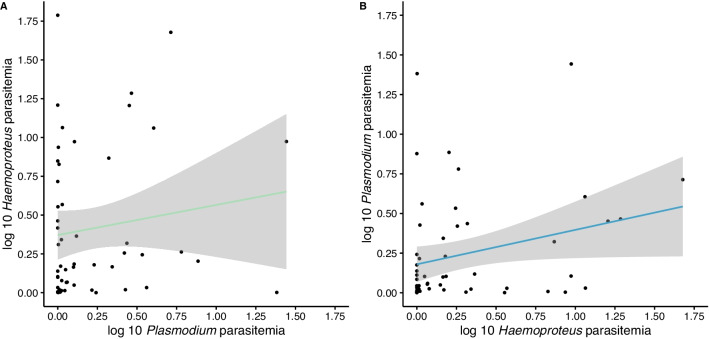
No correlated effects on parasitemia between ***H. passeris*** and ***P. relictum*** in juvenile house sparrows. The infection intensity (log 10 parasitemia) in juvenile house sparrows (**A**) blood positive with *H. passeris* (green) in relation with the infection intensity of *P. relictum* (log 10 parasitemia and also including non-infected individuals in this parameter, i.e. both blood positive and blood negative) and (**B**) blood positive with *P. relictum* (blue) in relation with the infection intensity of *H. passeris* (log 10 parasitemia and also including non-infected individuals in this parameter, i.e. both blood positive and blood negative). The positive correlations are non-significant and the grey area indicates the 95% confidence intervals (N = 11 juvenile house sparrows)

Then we did the same parasitemia analyses in the juveniles. The parasitemia of *H. passeris* in juveniles (including only data points when an individual was blood-positive for *H. passeris* in the analysis) differed between weeks (F = 7.93, df = 6, 35, P < 0.0001, Fig. 2), but there was no effect of the parasitemia of *Plasmodium* (P = 0.14), or individual identity (Δ2LL = 0, Fig. 4A). The parasitemia of *Plasmodium* in juveniles (including only data points when an individual was blood-positive *Plasmodium* in the analysis) was not affected by week (P = 0.17), by *H. passeris* parasitemia (P = 0.32), or by individual identity (Δ2LL = 0, Figs. 2 and 4B).

### Juvenile house sparrows have higher parasitemia than adults

**Fig. 5:**
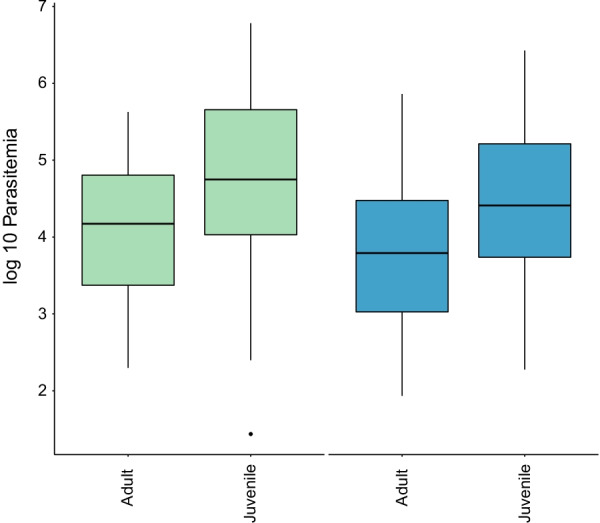
The parasitemias are significantly lower in adult compared with juvenile house sparrows. The infection intensity (log 10 parasitemia, box plot where the bottom and top of the box indicates the first and third quartiles, the central line corresponds to the median, the whiskers represent the highest and lowest value within 1.5 * the interquartile range respectively) in adult and juvenile house sparrows blood positive with *H. passeris* (green) and *P. relictum* (blue) from July until October 2016 (week 30–43, N = 19 adult and N = 11 juvenile house sparrows)

The parasitemia of *H. passeris* and *P. relictum*/*Plasmodium* was measured in both adult and juvenile sparrows from late July until early October 2016 (week 30–43). We used this subset of the data to compare parasitemia (including only individuals with parasitemias > 0) between age classes. Juveniles had significantly higher parasitemia than adults for *H. passeris* in this particular time period (F = 14.33, df = 1, 139, P = 0.0002, Fig. 5). The parasitemia varied between weeks (F = 6.83, df = 6, 139, P < 0.0001), but there was no effect of the random factor individual (Δ2LL = 0). For *P. relictum/Plasmodium*, the difference in parasitemia between adults and juveniles was significant in this particular time period (F = 7.52, df = 1, 136, P = 0.0069, Fig. 5), though not straightforward to interpret as there was a significant interaction between age and week (F = 2.87, df = 3, 136, P = 0.039), again without any effect of the random factor individual (Δ2LL = 0). Moreover, we cannot exclude that the putative mix of *Plasmodium* strains (SGS1 and PAGRI02) in juveniles, as indicated by Sanger sequencing, can have an impact on the analysis of the latter.

## Discussion

Both juvenile and adult sparrows were frequently co-infected with *H. passeris* and *P. relictum* and potential within-host interactions between these two haemosporidian parasites during their blood stages could therefore be studied in both age classes. The haemosporidian parasites in the juvenile sparrows in the present study are most likely primary infections, as indicated by the high parasitemia compared to the adults and the haemosporidian life cycle where the first peak parasitemia was reached after our sampling begun [32]. The haemosporidian parasites in adults on the other hand are likely to be relapses due to significantly lower parasitemias than in juveniles and the high prevalence of infections among all the studied adult birds. However, it cannot be completely excluded that some of the relapses are secondary infections since we only have used an observational approach to study co-infections of haemosporidian parasites, i.e. without vector-controlled regimes. Note, since all individuals carried both the haemosporidian parasites we were not able to test if the parasitemia differed between individuals with single infection and co-infection, but we could test to what extent the *H. passeris* and *P. relictum* / *Plasmodium* parasitemias co-varied in co-infections.

On sampling days when adult birds were blood-positive for *H. passeris*, their *H. passeris* parasitemia was higher when they also had high *P. relictum* parasitemia. Likewise, when adult birds were blood-positive for *P. relictum*, their *P. relictum* parasitemia was higher when they also had high *H. passeris* parasitemia. In both these cases the parasitemias were significantly positively correlated, thus, our data suggest facilitation, more specifically mutualism [14], between *P. relictum* and *H. passeris* during co-infections in adult house sparrows. Although the potential mechanisms behind such facilitation processes are unknown for birds and the interaction of the parasites cannot be fully verified [33], we suggest that the parasite preferences for RBCs, i.e. for young or mature age classes of RBCs, could be one factor driving the facilitation process. Facilitation through altered resource availability, i.e. an increased proportion of immature RBCs, has been shown previously in both human and rodent malaria [17, 20]. Moreover, resource availability has also been proposed to drive the interaction between avian malaria parasite species in experiments where birds were inoculated with two different avian malaria (*Plasmodium*) species [25, 26].

*Plasmodium relictum* parasites prefer to infect mature RBCs, which leads to anaemia and the production of new immature RBCs [26, 32]. This production of new immature RBCs could facilitate the presence of *H. passeris* if it prefers immature RBCs too. There is no data so far concerning preferences of RBC age classes by *H. passeris*, but a preliminary working hypothesis could be that gametocytes from *H. passeris* can infect also immature RBCs. Previous studies have shown that immature RBCs can be more metabolically active cells than mature RBCs [7], which could lead to a longer life span of the *H. passeris* gametocyte. Such a mechanism would explain why *H. passeris* may gain an advantage from *P. relictum*, i.e. commensalism, but does not include a mechanism of how *P. relictum* benefits from *H. passeris* infection. An alternative or additional, explanation to the positive associations between *H. passeris* and *P. relictum* parasitemias could be that there is variation in immune responses among house sparrow individuals [2], such that some house sparrow individuals can handle both *H. passeris* and *P. relictum* relatively well whereas other house sparrow individuals can handle neither parasite satisfactory.

In contrast to previous studies, we have studied co-infections between haemosporidian parasites belonging to different genera, *Plasmodium* and *Haemoproteus*, and not between species within *Plasmodium*. *Plasmodium* and *Haemoproteus* use the same tissue, bird blood, and competition for the limited resource RBCs could therefore have been a likely outcome. However, the parasitemia of both *P. relictum* and *H. passeris* is rather moderate in the house sparrows and intense competition for RBCs is therefore less likely. Co-infections with *P. relictum* and *H. passeris* are frequent among European house sparrows in southern Spain but their peaks in prevalence differs between the species. *Plasmodium relictum* has two yearly peaks in prevalence, late spring and early autumn, whereas *H. passeris* peak in the summer in the wild [35]. We cannot see such detailed temporal patterns in our limited experimental data but observed that sampling month had a significant effect on both infection status and parasitemia for both *P. relictum* and *H. passeris* in the adults.

Mutualism between *H. passeris* and *P. relictum* was not observed in juvenile house sparrows. The reason that we find mutualism in adult but not in juvenile sparrows could be due to differences in the immune response between the primary infection (acute phase in juveniles) and relapses (chronic phase in adults). For example, immune responses during the chronic phase are likely more specific than in the acute phase, and if it is difficult for the host to mount two different responses simultaneously, co-infecting parasites could benefit from the presence of each other. The lack of mutualism during co-infections in juveniles could simply be a result of lower statistical power, since we studied fewer individuals during a shorter time interval compared with adults.

Previous studies of co-infections of *Plasmodium* parasites in birds have inoculated two specific strains of avian malaria parasites to the bird host whereas we have screened for the prevalence of avian malaria in naturally infected bird hosts. *Haemoproteus passeris* reach higher infection intensities than *P. relictum* and in co-infections, and the detectability of *P. relictum* by Sanger sequencing of nested PCR-products is reduced. By running separate qPCRs for *Haemoproteus* and *Plasmodium* on every sample we overcome the methodological problem of *Haemoproteus* reducing the detectability of *Plasmodium*, but with our methodological design, we cannot exclude that some birds are infected with more than one *Plasmodium* strain/species. There is a high diversity of *Plasmodium* strains in house sparrows in southern Spain, although *P. relictum* is the dominating species [27, 30]. It is worth emphasizing the high repeatability (i.e. high variance among individuals) of *H. passeris* infection status and parasitemia in adult sparrows. In contrast, neither infection status nor parasitemia of *P. relictum* was repeatable in adults. This difference between *H. passeris* and *P. relictum* can be caused by differences in the parasites’ life cycles, where *Haemoproteus* species have their asexual stages (replication) restricted to internal organs, while *Plasmodium* species undergo asexual replication also in the blood [32].

Finally, several previous studies have reported higher parasitemia in juvenile birds compared to adults [36, 37], just in line with our findings in juvenile and adult house sparrows. However, a recent study comparing the parasitemia over the season in eight different bird species found substantial seasonal variation in both juveniles and adults but no overall difference in parasitemia between the age classes [38]. It is evident that several different parameters need to be considered when comparing parasitemia between juveniles and adults in natural populations and longitudinal sampling of individuals is a rewarding approach. Blue tits infected with *Haemoproteus majoris* often carry this parasite as a life-long chronic infection and the parasitemia decreases with age (at least until the birds are very old), suggesting that the ability to suppress the *Haemoproteus* parasites improve with age. We followed the variation in the *Haemoproteus* parasitemia in both juveniles and adults, however in only partly overlapping time intervals (adults between May and October and juveniles between August and October). The parasitemia decreased over the season in both age classes, with the highest parasitemia for adults in June and for juveniles in August. In August and September, when both age classes were sampled, juveniles have significantly higher parasitemia. Though, we cannot test if the peak parasitemia, in the putative relapse in the adults and in the primary infection in juveniles, differs among the age classes.

## Conclusions

Both adult and juvenile house sparrows were frequently co-infected by the two haemosporidian parasites *P. relictum* and *H. passeris*. In adult house sparrows there were reciprocal positive relationships in parasitemias, indicating either mutualism among the two parasites where both *P. relictum* and *H. passeris* gained from the presence of the other parasite or variation in immunocompetence among house sparrow individuals. The resulting higher parasitemia in co-infections is likely to result in stronger negative effects on host fitness during co-infection compared to single infections, though this remains to be tested in a suitable study system where haemosporidian parasites can be studied as both single- and co-infections.

## Methods

### Study site and experimental set up

Twenty-one adult wild house sparrows were captured with mist nets during February and June 2016 at the University Campus (38° 53′ 4.47″ N 07° 0′ 33.74″ W), and 15 juvenile house sparrows were captured in the surroundings of Badajoz city (38° 53′ 00″ N 06°58′ 00″ W) during June 2016. All birds were ringed with unique metal rings and then released into the aviaries located in the Experimental Garden at the University of Extremadura (38° 53′ 4.47″ N 07° 0′ 33.74″ W), southwest Spain. The aviaries have open-air conditions, and each cage is 3.5 × 1.5 × 2.5 m and contained up to eight individuals. Birds were provided with water and food ad libitum.

### Sample collection

Prior to the initiation of the study, birds were kept in the aviaries to achieve acclimatization of a minimum of two weeks. We monitored infection status and parasitemia of adult house sparrows during 18 months and of juvenile house sparrows during three months. Thus, blood samples from adults were collected monthly from late February 2016 to September 2017, whereas juvenile blood sampling occurred every second week from late July to late October 2016. We collected a single microcapillary tube (40 μl) of blood from the brachial vein and stored it in 500 ml of SET buffer (0.15 M NaCl, 0.05 Tris, 0.001 M EDTA, pH 8.0) until DNA extraction for molecular analyses. We also obtained a blood smear from each individual in order to estimate parasitemia (the proportion of infected RBCs) from microscopic examinations. Four juveniles and 11 adult sparrows died during the experiment. Once the study was completed, the surviving individuals were released in their original habitat.

### Molecular genetic screening of malaria parasites using Sanger sequencing

Haemosporidian parasites (*Plasmodium* and *Haemoproteus* spp.) were detected from blood samples using molecular methods. We used a standard ammonium acetate method [39] for DNA extraction. Genomic DNA was diluted to a concentration of 25 ng/µl for standard polymerase chain reaction (PCR). These samples were used as a template in a PCR assay for detection of the parasites using nested PCR protocols described by Waldenström et al. [40]. The amplification was evaluated by running 2.5 µl of the final PCR product on a 2% agarose gel. All PCRs contained one negative control for every eight samples. Positive amplifications were Sanger sequenced at the DNA sequencing facility, Department of Biology, Faculty of Science, Lund University on a ABI PRISM 3130 genetic analyser (Applied Biosystems, New Jersey, USA). The obtained sequences were then compared against the MalAvi database [31] using BLASTn to identify the parasite lineages. The haemosporidian parasites in each sparrow individual were DNA sequenced repeatedly, using samples from several different time points (Additional file 1).

### Quantification of malaria parasites

Blood samples were fixed in absolute methanol and stained with Giemsa as described by Valkiūnas et al. [41]. Approximately 100 fields were examined at low magnification (400x) and then at least 30 fields were studied at high magnification (1000x). The parasitemia was estimated as a percentage by actual counting of the number of parasites per 1000 erythrocytes [42]. One sample each with single infections of relatively high parasitemia, 0.98% for *Haemoproteus* and 2.9% for *Plasmodium*, were selected as reference samples for the real-time quantification PCR (qPCR) analysis. All samples were diluted to a DNA concentration of 1 ng/µl for quantification of parasitemia by qPCR. We used an Mx3005 real-time PCR instrument (Stratagene, USA) with SYBR-Green based detection (Platinum SYBR Green qPCR SuperMix-UDG; Invitrogen, USA) according to the manufacturer’s protocol. Given that DNA content varies around the target 1 ng/µl across samples, a first reaction was carried out with the host-specific primers SFSR/3Fb and SFSR/3Rb (Table 2) [43] to accurately measure the content of host DNA in the samples. For quantification of host DNA, each plate contained a standard curve based on a two-fold serial dilution of a non-infected sample with a starting DNA concentration of 4 ng/µl. In order to estimate parasitemia separately for *Haemoproteus* and *Plasmodium*, we used previously published *Plasmodium*-exclusive primers PQR/PQF [44] and designed a pair of *Haemoproteus*-exclusive primers *Cytb*734F/*Cytb*857R (Table 2). The proposed genus-specific amplification of the primers was checked against an alignment containing sequences of all *Plasmodium* and *Haemoproteus* lineages commonly found in house sparrows [28]. All qPCR reactions started with a pre-incubation at 50 °C (2 min) to remove potential PCR contaminants by uracil-n-glycosylase digestion followed by a denaturation at 95ºC (2 min). The temperature profile consisted at 43 cycles of 94 °C for 30 s, 52–57 °C (Table 2) for 45 s and 72 °C for 45 s, followed by a melting analysis between 48 and 95 °C. All samples were run in duplicates and DNA copies were scored as the average across the two wells. For quantification of parasites, the *Haemoproteus* and *Plasmodium* reference samples (see above) were included on each plate to adjust for inter-plate variation. Finally, six negative controls were included per plate and the results were only accepted when the negative controls yielded no specific amplification. At the end of each run, the amplification curves were inspected to obtain Ct values (the number of cycles when the florescence signal reached the automatically generated threshold line) for each sample and the melting curves were checked to detect false positive (i.e. non-specific amplifications). The parasite quantity of each sample (x) was calculated relative the reference sample (r) using the ΔCt method (2^Ctr − Ctx^) and converted to relative parasitemia by multiplying the ΔCt values with the microscopic estimates of parasitemia of the reference samples. These relative parasitemia values were then corrected for variation in the amount of host DNA, by dividing the values of the qPCR estimates with the host DNA concentration. Finally, the estimates were transformed to logarithm in base 10 (log10) for statistical analyses.

**Table 2:** Primers used for the qPCR

Target	Primer name	Sequence (5’-3’ direction)	Annealing temperature	Product size (bp)
*Haemoproteus*	*Cytb*734F	ATGCAATTGTAGTAGATAGATATGT	56	124
	*Cytb*857R	GCTATCATAACTAATAAACCAGC		
*Plasmodium *[44]	PQF	CATGGATTTGTGGTGGATATCTTG	52	198
	PQR	TATCRAGACTTAAWAGATTTGGATAGAAG		
Host [43]	SFSR/3Fb	ACTAGCCCTTTCAGCGTCATGT	57	114
	SFSR/3Rb	CATGCTCGGGAACCAAAGG		

### Statistical analyses

To test for interactions between *P. relictum* and *H. passeris*, we used a similar statistical approach as Lello et al. [10]. Thus, to test for effects of *P. relictum* on *H. passeris* infection status, we performed a logistic regression with presence/absence of *H. passeris* in blood as dependent variable, and *P. relictum* parasitemia (including samples with zero parasitaemia) as predictor. To test for effects of *P. relictum* on *H. passeris* parasitemia, we performed a glm with *H. passeris* parasitemia (only samples with > 0 parasitemia) as dependent variable, and *P. relictum* parasitemia (including samples with 0 parasitemia) as predictor. Corresponding analyses were performed to investigate the effect of *H. passeris* on *P. relictum.* All analyses were done separately for adults and juveniles since several previous studies have shown that both infection status and parasitemia differs between age classes for haemosporidian parasites [45]. For infection status in adults we performed generalized linear mixed models [proc glimmix in SAS 9.4 (SAS Institute, NC, USA); method = Laplace; dependent variable: infection status of either *H. passeris* or *P. relictum* (presence/absence); fixed factors: year, month and their interactions; covariate: parasitemia of either *H. passeris* (in the analysis of *P. relictum* infection status) or *P. relictum* (in the analysis of *H. passeris* infection status); random factor: individual]. For parasitemia in adults we performed general linear mixed models (proc mixed; dependent variable: parasitemia of either *H. passeris* or *P. relictum*; fixed factors: year, month and their interactions; covariate: parasitemia of either *H. passeris* or *P. relictum*; random factor: individual). For juveniles, we performed similar analyses but with week as factor instead of year and month (since juveniles were only sampled during one year). Finally, differences in parasitemia were also tested in adults and juveniles, combined for week 30–43 (July to October) 2016, where we had data from both age classes. We again performed general linear mixed models (proc mixed; dependent variable: parasitemia of either *H. passeris* or *P. relictum*; fixed factors: week, age and their interaction; random factor: individual). Non-significant terms were deleted in a stepwise manner (interactions first) at P > 0.1. P-values for fixed effects were determined by F tests (type 3). Denominator degrees of freedom were determined by Satterthwaite approximation. Plots were made with the R package ggplot2 (v. 2.1.0) [46].

## Supplementary information

**Additional file 1.** Identification of Haemosporidian parasites (*Haemoproteus passeris* (PADOM05), *Plasmodium relictum* (SGS1, GRW11), and *Plasmodium spp* (PAGRI02)) in house sparrows by Sanger sequencing. Haemosporidian parasites in bird blood in both adult and juvenile house sparrows identified by Sanger sequencing (Sanger sequencing identified three main parasite species: *Haemoproteus passeris *(PADOM05), *Plasmodium relictum* (SGS1, GRW11), and *Plasmodium spp *(PAGRI02)) [30]. Every individual was sampled repeatedly over the season(s) (Year and Date).

**Additional file 2.** Quantification of Haemosporidian parasites (*Haemoproteus spp* and *Plasmodium spp*) in house sparrows using qPCR. Haemosporidian parasites in bird blood in both adult and juvenile house sparrows quantified by qPCR (*Haemoproteus spp* (HQ) and *Plasmodium spp* (PQ)). Every individual (1-35) was sampled repeatedlyover the season(s) (Year, Week and Date). The adult individuals 2 and 20 only had single infections, three juveniles were not infected and one juvenile was only sampled twice, and these six individuals were excluded from further analyses.

**Additional file 3.** Seasonal changes in infection status and parasitemia in adult house sparrows, nine individuals. Changes in infection status (% blood-positive individuals) and infection intensity(log10 parasitemia, box plot where the bottom and top of the box indicates the first and third quartiles, the centralline corresponds to the median, the whiskers represent the highest and lowest value within 1.5 * the interquartile range respectively) for *H. passeris* (green bars) and *P. relictum* (blue bars) in adult house sparrows, from February 2016 to September 2017, when studied in nine individuals over an 18-month period (note a lower sample size at the last sampling point in September 2017 (N=6)).

## Data Availability

All data generated or analysed during this study are included in this published article [and its additional files], see Additional file 1 and Additional file 2. Note that the chromatograms from Sanger sequencing (Additional file 1) only were used for confirming presence/absence of Haemosporiadian parasites (H. passeris and P. relictum in particular) by comparisons with the Malavi database [31]. The accession numbers to the three dominant malaria parasites found are SGS1 - AF495571, PADOM05 - HM146898 and PAGRI02 - JX196865.
