# Supplementary figures and images for: Reciprocal positive effects on parasitemia between coinfecting haemosporidian parasites in house sparrows
Source: BMC Ecol Evol. 2022 Jun 2;22:73. doi: 10.1186/s12862-022-02026-5 (PMC9164529; doi:10.1186/s12862-022-02026-5)

Haemoproteus Plasmodium

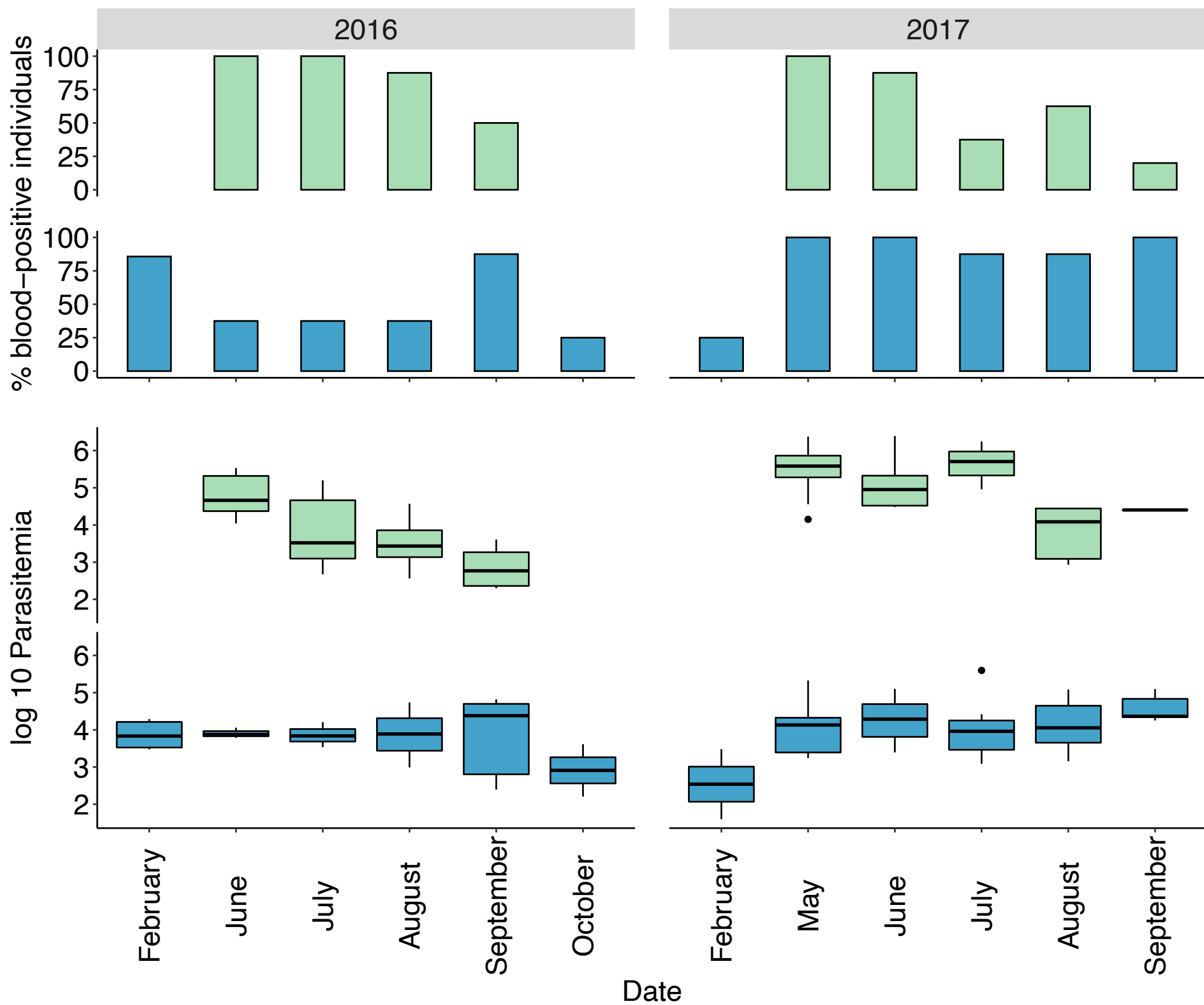

Supplement: Supplementary file 3 — Additional file 3. Seasonal changes in infection status and parasitemia in adult house sparrows, nine individuals. Changes in infection status (% blood-positive individuals) and infection intensity(log10 parasitemia, box plot where the bottom and top of the box indicates the first and third quartiles, the centralline corresponds to the median, the whiskers represent the highest and lowest value within 1.5 * the interquartile range respectively) for H. passeris (green bars) and P. relictum (blue bars) in adult house sparrows, from February 2016 to September 2017, when studied in nine individuals over an 18-month period (note a lower sample size at the last sampling point in September 2017 (N=6)). [file 12862_2022_2026_MOESM3_ESM.pdf]
